# Juvenile trabecular ossifying fibroma: Immunohistochemical expression of MDM2, CDK4 and p53 compared to conventional ossifying fibroma

**DOI:** 10.4317/jced.59116

**Published:** 2022-01-01

**Authors:** Nikolaos G. Nikitakis, Maria Georgaki, Stavroula Merkourea, Risa Chaisuparat, Gary Warburton, Marcio A. Lopes, John C. Papadimitriou, Robert A. Ord

**Affiliations:** 1 Department of Oral Medicine & Pathology and Hospital Dentistry, School of Dentistry, National and Kapodistrian University of Athens, Greece; 2 Department of Oral Pathology, Faculty of Dentistry, Chulalongkorn University, Bangkok, Thailand; 3 Department of Oral and Maxillofacial Surgery, Dental School, University of Maryland, Baltimore, USA; 4 Department of Oral Diagnosis, Piracicaba Dental School, University of Campinas, Brazil; 5 Department of Pathology, Medical School, University of Maryland, Baltimore, USA

## Abstract

**Background:**

Juvenile ossifying fibroma (JOF) is an uncommon benign fibro-osseous lesion of the craniofacial skeleton; compared to conventional ossifying fibroma (OF), JOF is characterized by local aggressiveness and propensity for recurrence. The biologic basis for this different biologic behavior between JOF and OF remains elusive. The aim of this study was to evaluate the immunohistochemical expression of MDM2, CDK4 and p53, molecules associated with bone oncogenesis, in the trabecular variant of JOF.

**Material and Methods:**

The study material consisted of five cases of trabecular JOF, affecting three male and two female patients with a mean age of 11.8 years. Three cases arose in the maxilla and two in the mandible. All cases were initially treated by enucleation; two cases recurred necessitating more aggressive treatment. Immunohistochemical study of MDM2, CDK4 and p53 was performed in all cases, as well as in five control cases of conventional OF.

**Results:**

CDK4 positivity was noted in all JOF cases; the staining pattern was diffuse and strong in 4 cases and focal and weak in one case. In contrast, 4 out of 5 cases of OF were weakly and focally CDK4 positive, the remaining one being negative. Immunostaining for MDM2 was observed in 3 JOF cases; all OF were MDM2 negative. All cases of OF and JOF were negative for p53, except for one focally positive JOF case.

**Conclusions:**

CDK4 and MDM2 expression in the trabecular variant of JOF is higher compared to conventional OF. In contrast, p53 expression is almost universally negative in JOF and OF. Despite some overlapping features, differential expression patterns of proteins involved in bone oncogenesis can elucidate the pathogenesis and may facilitate accurate diagnosis and prediction of behavior of bone tumors in the craniofacial region.

** Key words:**Juvenile ossifying fibroma, trabecular variant, conventional ossifying fibroma, MDM2, CDK4, p53.

## Introduction

Juvenile ossifying fibroma (JOF) was introduced in the WHO classification of odontogenic tumors in 1992, defined as a fibro-osseous lesion of the craniofacial skeleton affecting children under the age of 15 years (1). Two subtypes of JOF with distinct clinicopathologic features have been described: the trabecular variant tends to occur in the jaws of younger individuals (mean age range: 8.5-12 years), whereas the psammomatoid variant affects a wider age range (mean age: 20 years) with a propensity for extragnathic locations, predominantly the sinonasal and orbital bones (2-4). Although a benign neoplasm, JOF behaves as a locally aggressive lesion with a high recurrence rate ranging from 30-56% (2-4).

The advent of molecular biology has increased our awareness of the mechanisms of tumorigenesis, including bone oncogenesis. Various molecules have been investigated in the context of bone neoplasms, especially in osteosarcoma (OS) and its variants and mimics, including the well-characterized oncogenes CDK4 and MDM2 and the tumor suppressor gene p53 (5-8). In a previous study, we have analyzed the expression of these genes located on chromosome 12q13-15, in osteosarcoma (OS) of the jaws; their high expression supported the notion that p53-MDM2 and Rb-cyclin D–CDK4 pathways play a role in the pathogenesis of these gnathic tumors (9). However, the molecular features of JOF, including the protein expression of the aforementioned genes, and their similarities and differences with OS and the more indolent conventional ossifying fibroma (OF) remain largely unknown.

In this study, we present the clinicopathologic features of five trabecular-type JOF cases affecting the jaws and analyze their immunohistochemical profile for bone tumorigenesis-related molecules CDK4, MDM2 and p53 in comparison with conventional OF.

## Material and Methods

-Study material

Five JOF of the jaws were retrieved from the medical records of the Department of Oral and Maxillofacial Surgery, University of Maryland, Baltimore. Information about age, gender, race, location of lesion, treatment modalities, recurrence, follow-up and current status was recorded. In addition, five control cases of conventional ossifying fibroma (OF) were included. Microscopic slides and pertinent clinical and imaging information were reviewed, and the histopathologic diagnosis was confirmed in all cases.

-Immunohistochemical analysis 

Five JOF and five OF were stained with a standard immunohistochemical protocol used and described in our previous study (9). Briefly, all specimens underwent short time decalcification (24h) prior to paraffin inclusion, as they consisted mostly of soft tissues. Five micron thick-sections of paraffin-embedded tissues were mounted on glass slides, deparaffinized and rehydrated. The slides were placed in Citra-solution (Biogenex CA-USA, HK086-9K) and treated by two cycles. The endogenous peroxidase activity and non-specific protein were blocked. After extensive washing, the sections then were incubated for 1 hour with primary antibody: MDM2 (Santa Cruz Biotechnology, SMP14, sc-965) 1:50, CDK4 (Santa Cruz Biotechnology, C-22, sc-260-G) 1:50, p53 (Biogenex, CA-USA, MU195-UC) 1:50. The sections then were incubated with secondary antibody for 30 minutes (Biogenex CA-USA, HK268-UK for MDM2 and p53 and Biogenex CA-USA, HK327-UG for CDK4) and for another 30 minutes with a StrepABComplex/HRP (Dako, Denmark, K0377). The specimens were stained with diaminobenzidine and counterstained with Harris hematoxylin. OS cases from our previous study (which has been performed using the aforementioned immunohistochemical procedure and the same antibodies) were used as positive controls (9). Negative controls were treated identically with omission of the primary antibody.

The immunostains were evaluated by two pathologists (NN and RC), according to the percentage of positive cells and the intensity of the staining, according to the following scales (10):

Percentage of positive cells: - (0-5% positive cells), + (6-25% positive cells), ++ (26-50% positive cells) and +++ (51-100% positive cells) and Intensity of staining: weak (W), moderate (M), strong (S).

## Results

-Demographic, clinicopathologic, treatment and follow-up findings of JOF cases (Table 1) 

**Table 1:**
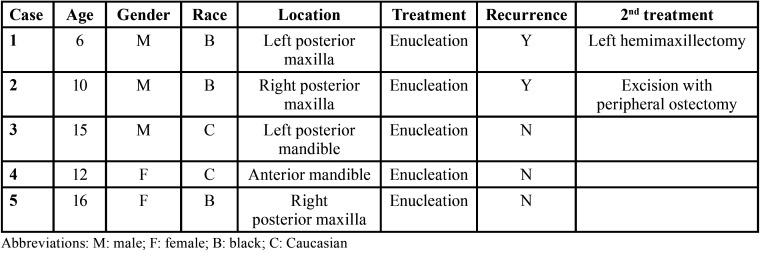
Demographic and clinical data of JOF cases.

The mean age of the five JOF patients was 11.8 years, ranging from 6-16 years, with a male to female ratio of 3:2. Three patients were African American and two were Caucasian. Three cases occurred in the posterior maxilla and two cases in the mandible (one in posterior and one in anterior location).

All JOF lesions presented as a painless bony expansion; in two lesions, increased vascularity was noted on the overlying mucosa. The mean reported duration of the lesions prior to diagnosis was 2.6 months, ranging from 2 to 3 months.

Imaging examination revealed all lesions to present as mixed areas of lucency and opacity with well-defined borders, associated with tooth displacement and bony expansion. In two lesions, the adjacent teeth exhibited widened periodontal ligament spaces. The size of the lesions ranged from 2.0 to 8.0 cm.

**Figure 1:**
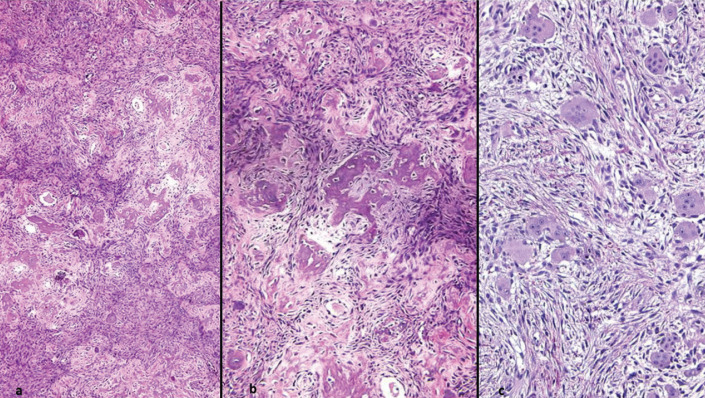
Photomicrogaphs of JOF trabecular variant showing a cellular fibrous stroma containing a mineralized component in the form of bone trabeculae (a, b) and focal accumulation of multinucleated cells (c) (a: H&E 100X; b and c: H&E 200X).

Microscopically, all five JOF cases exhibited typical features of the trabecular variant (Fig. 1). Histopathologic examination of all tumors revealed cellular fibrous stroma containing a mineralized component in the form of bone trabeculae and irregular strands of highly cellular osteoid, encasing plump osteocytes and lined by osteoblasts. Focal accumulations of multinucleated giant cells were observed in three cases.

All five JOF cases were initially treated by enucleation and curettage. Of these, three did not show any evidence of recurrence for a mean follow up period of 10 months. The remaining two cases recurred at 6 and 7 months and were managed with hemimaxillectomy or peripheral ostectomy, respectively.

-Demographic, clinicopathologic, treatment and follow-up findings of control OF cases

The five patients with conventional OF cases (used as controls) had a mean age of 38 years (ranging from 20-55 years), with a male to female ratio of 2:3. Three patients were Caucasian and two were African American. All cases occurred in the mandible, four in posterior and one in anterior location.

Four lesions presented as a bony expansion, and one lesion was discovered as an incidental finding on routine dental radiograph. On clinical examination, there were no abnormalities noted in the overlying mucosa in any of the patients. In two cases with available information, the duration of the lesions was reported as 3 and 7 years, respectively.

Imaging studies revealed four lesions to be mixed radiolucent-radiopaque, while one lesion was predominantly sclerotic; all lesions had well defined borders. Neither tooth displacement nor widening of the periodontal ligament was observed in any case. The size of the lesions ranged from 2.0 to 7.0 cm.

All lesions were treated by enucleation and curettage. No recurrences were noticed in any patient after a follow-up period ranging from 1-6 years.

-Immunohistochemical results (Table 2)

**Table 2:**
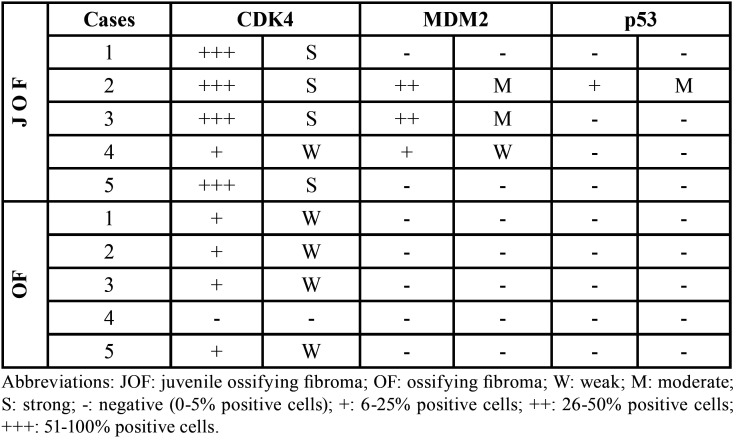
Immunohistochemical expression of CDK4, MDM2 and p53 in JOF and OF.

CDK4 staining 

**Figure 2:**
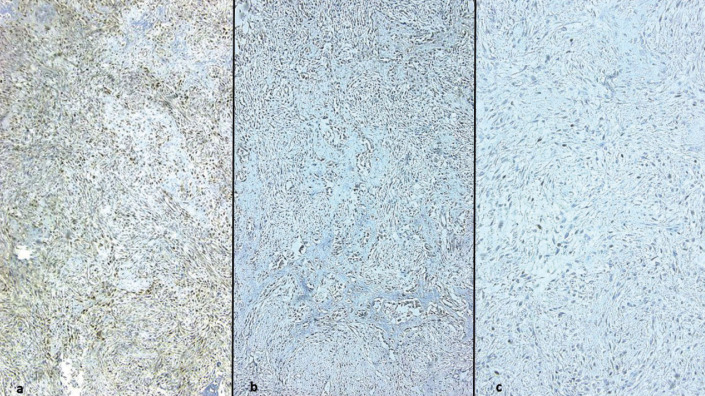
a) Immunostaining for CDK4 in a JOF case showing a diffuse and strong staining pattern. b) Moderate MDM2 immunoexpression in a JOF case. c) Focal p53 immunoexpression in a JOF case (400X).

Immunohistochemical analysis revealed that CDK4 was positive in all JOF cases (Fig. 2a). The staining pattern was diffuse and strong in four cases and focal and weak in one case. In contrast, four cases of conventional OF were weakly and focally CDK4 positive, while the remaining one case was negative.

MDM2 staining 

MDM2 positivity was noted in three JOF cases (Fig. 2b). There was moderate intensity in 21-50% of cells in two cases (also showing diffuse and strong CDK4 positivity). One case demonstrated weak staining in less than 20% of cells (accompanied by focal and weak CDK4 immunoreactivity); in contrast, all OF were negative for MDM2.

p53 staining 

All OF and JOF cases were negative for p53, except one focally positive JOF case (Fig. 2c).

**Figure 3:**
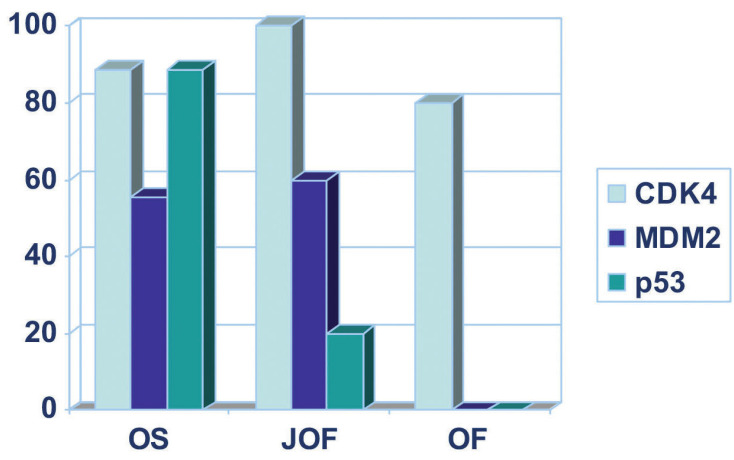
Bar graph demonstrating the percentage of positive JOF and OF cases for CDK4, MDM2 and p53. For comparison, the percentage of OS cases demonstrating positivity for the same markers is also included, based on previously published data by our group (9).

The percentage of positive JOF and OF cases for CDK4, MDM2 and p53, in comparison with the percentage of OS cases based on previously published data by our group (9), is presented in Fig. 3.

## Discussion

According to the latest 2017 WHO classification of head and neck tumors, JOF is a clinicopathologic variant of ossifying fibroma OF (11), characterized by rapid growth, potential of local invasiveness and tendency to recur (3). JOF diagnosis necessitates clinicopathologic correlation, taking into account various parameters, such as age of onset, location, clinical and radiologic features, microscopic characteristics and biologic behavior (2,3,12-15). Two subtypes of JOF with differences in their clinicopathologic features are described in the literature: juvenile trabecular ossifying fibroma (JTOF) and juvenile psammomatoid ossifying fibroma (JPOF) (2,3,11). In the present study, all cases were classical examples of JTOF.

The diagnosis of JTOF is not always straightforward. As for all fibro-osseous, a combination of clinical, radiologic and microscopic findings is required for final diagnosis. The main entities that enter the differential diagnosis are other fibro-osseous lesions, including conventional OF, as well as malignant tumors, especially OS. Although careful evaluation of the specific clinicopathologic features allows distinction of JOF in most cases, borderline cases may pose significant diagnostic difficulties. Therefore, the elucidation of the molecules and pathways involved in the pathogenesis of JTOF may contribute to the discovery of markers that can distinguish it from its benign and malignant counterparts. 

Altered cell cycle and apoptosis regulation leads to tumorigenesis. Several lines of evidence have shown that the dysregulation of p53-MDM2 and Rb-Cyclin D-CDK4 pathways, which regulate the G1-S phase of cell cycle, is involved in tumorigenesis of OS (5-8), including its gnathic counterparts (9,16-18).

p53, one of the most widely studied tumor suppressor genes in humans, is a transcription factor, encoded by TP53 gene, that regulates genes involved in cell cycle, DNA damage response and apoptosis (19). Expression of mutated p53 has been associated with poor prognosis and response to chemotherapy in many human malignancies (20,21). p53 mutations are also frequently detected in OS and have been suggested as possible targets of novel therapies (22). p53 has been also found to be overexpressed in a significant subset of OS of the jaws (9,16,23,24) Specifically, p53 positivity was detected in 88% and 52% of the jaw OS cases reported by Lopes *et al*. (9) and Junior *et al*. (16), respectively, and has been found to be comparable between OS of the jaws and long bones (23); however, a correlation between p53 expression and jaw OS prognosis has not been established (24). Despite its role in malignant bone tumors, the possibility that p53 aberrations may participate in the oncogenesis of benign bone tumors of the jaws, including OF and JOF, has not been investigated. Although confirmation requires a larger sample, our data, showing focally moderate p53 immunostaining in 1 out of 5 JOF cases and lack of immunostaining in 5 OF cases, indicate that p53 aberrations are not involved in OF pathogenesis, while their presence and participation in the oncogenesis of a subset of JOF cases cannot be excluded.

MDM2 is an E3 ligase involved in transport of p53 to the cytoplasm for its degradation (25). Gene amplification of MDM2 has been described as the pathway of tumorigenesis or tumor progression in various sarcomas, including OS (26). CDK4 is a cyclin-dependent kinase implicated in G1 to S cell cycle transition through phosphorylation and inactivation of the retinoblastoma tumor suppressor protein (pRb) (27). Amplification of the chromosomal region 12q13-15, involving both MDM2 and CDK4, is a frequent event in OS, resulting in the overexpression of the corresponding proteins (8). MDM2 and CDK4 amplification and overexpression is especially common in low-grade OS, including their dedifferentiated counterparts (28). In particular, co-amplification of MDM2 and CDK4 is more prevalent in the low-grade parosteal OS (67%) compared to high-grade classical OS (12%) (8), showing a correlation between amplification levels and tumor grading and progression in the former group (29). Noticeably, CDK4 amplification and overexpression may serve as a useful predictive biomarker of chemoresistance in patients with OS (30).

From a diagnostic standpoint, evaluation of MDM2 and CDK4 by immunohistochemistry has been proposed as a useful tool in distinguishing low-grade OS, including both central and parosteal, from benign fibro-osseous lesions with similar microscopic features (6,7). Dujardin *et al*. (6) noticed that 89% of 72 low-grade OS cases were positive for MDM2 and/or CDK4 immunostaining, while all 107 cases of fibrous or fibro-osseous lesions of the bone (including 6 maxillofacial OF) or para-osseous soft tissue and 20 control cases of conventional high-grade OS were negative. Yoshida *et al*. (7) explored the use of MDM2 and CDK4 immunohistochemistry for the histologic diagnosis of low-grade OS: all 23 low-grade OS cases expressed one or both markers (100%), with 13 cases (57%) expressing both; in contrast, only 1 out of 40 benign mimics (including fibrous dysplasias, myositis ossificans and other entities, but not OF) was immunohistochemically positive for MDM2 or CDK4.

MDM2 and CDK4 amplification and overexpression have been also shown in gnathic OS (9,16-18), but their investigation in benign fibro-osseous lesions of the jaws, including JOF and conventional OF, has been very limited.

MDM2 immunohistochemical expression in OS of the jaws has been detected in 55.5% and 24% of cases studied by Lopes *et al*. (9) and Junior *et al*. (16), respectively; however, no control cases of benign fibro-osseous lesions were included in these two studies. On the other hand, Guerin *et al*. (17) found that only 3 out of 36 cases of mandibular OS cases were immunohistochemically positive for MDM2 (two of them also showing MDM2 amplification by qPCR, the third one being non-evaluable), classified as differentiated/dedifferentiated OS. Noticeably, a control group of benign fibro-osseous lesions, including 15 OF, was uniformly negative for MDM2 overexpression. In the same study (17), a distinct molecular signature, i.e. MDM2 and RASAL1 amplification without MDM2 overexpression, was identified in a small subset of giant cell-rich high grade mandibular OS, which contained areas mimicking JOF. The authors suggested that JOF cases exhibiting this amplification may be related to, or even at risk for transformation into, high grade OS, requiring closer follow-up and aggressive management. Similarly, Tabareau-Delalande *et al*. (31) reported an association between chromosome 12 long arm rearrangement covering MDM2 and RASAL1 genes with aggressive craniofacial JOF. Specifically, MDM2 amplification was detected in 69% of JOF (70% in trabecular and 67% in psammomatoid variants), as opposed to only 6% of conventional OF; however, this amplification was not accompanied by immunohistochemical overexpression. Interestingly, simultaneous amplification of MDM2 and RASAL1 was significantly more prevalent in JOF compared with conventional OF and other benign craniofacial fibro-osseous lesions, while JOF cases harboring amplification tended to exhibit a locally aggressive or recurrent behavior. This chromosome rearrangement was the first recurrent molecular abnormality to be reported in JOF (31). More recently, Limbach *et al*. (18) reported MDM2 immunohistochemical positivity and gene amplification in 63% (7 out of 11) and 25% (1 out of 4) of craniofacial OS, respectively; in contrast, all 14 studied craniofacial benign fibro-osseous lesions (including three central OF, but not JOF) were negative for MDM2 immunohistochemistry. Based on a comprehensive literature review, the same authors (18) reported that 33% (28 of 35) of craniofacial OS were MDM2 positive on immunohistochemistry, out of which 46% (13 of 28) demonstrated MDM2 amplification, while all studied craniofacial benign fibro-osseous lesions (including OF, but not JOF) were immunohistochemically negative for MDM2 (although MDM2 staining was noticed in associated osteoclast-like giant cells in both benign and malignant lesions). Similarly, in the present study, all studied jaw OF cases were negative for MDM2; however, 3 out of 5 JOF showed mild or moderate positivity for JOF of the jaws. To the best of our knowledge, this is the first time that MDM2 immunohistochemical positivity is reported in JOF, indicating that, in contrast with conventional OF and other fibro-osseous lesions of more indolent behavior, aberrations in MDM2 may play some role in pathogenesis and aggressive behavior of JOF.

Several of the aforementioned studies have also investigated CDK4 immunohistochemical profile in benign and malignant jaw bone lesions. Specifically, CDK4 was highly expressed in jaw OS in the studies of Lopes *et al*. (9) (56%) and Junior *et al*. (16) (84%). Similarly, Limbach *et al*. (18) found that 7 out of 11 (63%) of their craniofacial OS were immunohistochemically positive for CDK4; based on their comprehensive literature review, it was found that the majority of studied craniofacial OS show CDK4 immunohistochemical expression (35 out of 49 cases, 71%), as well as CDK4 amplification (7 out of 10, 70%). On the contrary, all 14 craniofacial benign fibro-osseous lesions (including three central OF) studied by Limbach *et al*. (18), as well as the 10 maxillofacial benign fibro-osseous lesions (including 6 OF) studied by Dujardin *et al*. (6), were CDK4 negative; however, no JOF cases have been investigated before for CDK4 expression. In our study, all JOF cases and the majority of OF cases were positive for CDK4; however, there was a noticeable difference in the percentage and intensity of CDK4 immunopositivity, with the majority of JOF cases (4 out of 5) exhibiting strong and diffuse staining as opposed to the focal and weak expression pattern in OF. These data suggest that CDK4 may participate in JOF oncogenesis and its overexpression may be linked to its locally aggressive behavior compared to the more indolent clinical course of a conventional OF.

Conclusively, the expression profile of p53, MDM2 and CDK4 seems to show considerable differences between JOF and OF. The CDK4 is expressed in both JOF and OF, although the latter shows diminished (or even absent) staining. MDM2 expression is found only in a subset of JOF, being universally absent in conventional OF. Finally, p53 expression is almost absent in both lesions, with only one case of JOF showing focal positivity. The distinct immunohistochemical profiles demonstrated by JOF and OF in the present study seem to correspond to their different clinical behavior. Similar to its aggressive but benign biologic behavior, JOF immunoprofile for the investigated molecules appears to be intermittent to those of OF and OS. Furthermore, differences in MDM2, CDK4 and p53 staining may be employed in the differential diagnosis of JOF and OF, as well as in their discrimination from OS. Future studies based on larger samples should better establish the potential diagnostic usefulness of these markers and explore their prognostic and predictive significance.
